# Civil and forensic patients in secure psychiatric settings: a comparison

**DOI:** 10.1192/pb.bp.115.052910

**Published:** 2017-06

**Authors:** Nuwan Galappathie, Sobia Tamim Khan, Amina Hussain

**Affiliations:** 1 St Andrew's Healthcare, Birmingham, UK

## Abstract

**Aims and method** To evaluate differences between male patients in secure psychiatric settings in the UK based on whether they are detained under civil or forensic sections of the Mental Health Act 1983. A cohort of patients discharged from a secure psychiatric hospital were evaluated for length of stay and frequency of risk-related incidents.

**Results** Overall, 84 patients were included in the study: 52 in the forensic group and 32 in the civil group. Civil patients had more frequent incidents of aggression, sex offending, fire-setting and vulnerability, whereas forensic patients had more frequent episodes of self-harm.

**Clinical implications** Secure hospitals should ensure treatment programmes are tailored to each patient's needs. Civil patients require greater emphasis on treatment of their mental illness, whereas forensic patients have additional offence-related treatment needs. Regular liaison between forensic and general adult services is essential to help ensure patients can return to appropriate settings at the earliest opportunity in their recovery.

Further to the closure of the asylums and subsequent rare but high-profile failures in community care, forensic psychiatry in the UK has rapidly expanded, with the development of high, medium and low secure in-patient services across the country as well as specialist forensic mental health teams in the community.^1^ Alongside its expansion, an ongoing debate related to its interface with general psychiatry has persisted.^2^ In England and Wales, the forensic *v*. general psychiatry divide extends to the subdivision of in-patients detained under the Mental Health Act 1983 into those affected by civil and forensic sections. Patients detained under Part II of the Act are termed ‘civil patients’. They can be detained under Section 2 for assessment and treatment for up to 28 days where there is suspicion of a mental disorder. Section 2 can be converted to Section 3 for further treatment. Alternatively, patients can be admitted directly under Section 3 when there is a known mental disorder. Patients detained under Part III of the Act are termed ‘forensic patients’, given their involvement in the criminal justice system through the courts and prisons.

While forensic units have expanded, there has been an overall reduction of in-patient bed numbers in the UK, which have fallen from 155 000 in 1954 to just 18 166 as of 31 March 2014. The number of patients detained in all settings under civil sections during 2013/2014 was 32 781, of which 25 300 were under Section 2 and 7481 were under Section 3. During 2013/2014 there were 1847 detentions under forensic sections: 99 under Section 35 or 36, which is admission for assessment or treatment via the courts; 763 under Section 37 hospital orders, allowing detention in hospital instead of a prison sentence; and 457 under Section 47, which allows transfer of a serving prisoner to hospital.^2,3^

Secure psychiatric hospitals are generally geared towards providing assessment, treatment and rehabilitation for forensic patients, since they are the majority group in such hospitals. In particular, Coid *et al* found that 69% of patients in medium security were detained under forensic sections.^4^ Despite this discrepancy in distribution, research into whether there are differences between these groups is limited. The only study we could identify was that by Reed (2004),^5^ who evaluated the differences between civil and forensic in-patients in a low secure intellectual disability setting and found, surprisingly, that the forensic patients were less likely to be aggressive or use weapons but more likely to harm themselves.^5^ It is not known whether these findings are isolated to intellectual disability settings. Therefore, we present findings from our evaluation of male patients discharged from a secure psychiatric hospital (excluding intellectual disability – the hospital does not cater for such patients) and suggest recommendations on how to meet the differing clinical needs identified in each group.

## Method

The study was conducted as part of a service evaluation into length of stay at St Andrew's Healthcare, Birmingham, and registered with the St Andrew's Clinical Audit Team. In keeping with previous similar evaluations, ethical approval was not required as the study evaluated retrospective, non-patient-identifiable data from health records as part of service evaluation.^5^ Data were retrospectively collected for all discharges from the two medium and three low secure wards since the opening of the hospital in March 2009 to the study end point of 30 December 2014. The source of data were patients' electronic health records, including medical reports, Historical Clinical Risk Management-20 (HCR-20) assessments, Care Programme Approach records, electronically recorded risk incident logs and discharge summaries.

Summary statistics were calculated for all patients evaluated. Patients were then grouped by whether they were initially detained under a civil or forensic section at the start of their admission to St Andrew's Birmingham. In order to evaluate illness severity between the two groups, Health of the Nation Outcome Scales for Users of Secure and Forensic Services (HoNOS-secure) assessment scores taken at admission and discharge were noted.^6^ A power calculation was not performed but all available data were used in the analysis. The average length of stay was calculated for each group. SPSS version 16 for Windows was then used to calculate independent *t*-test statistics to examine any between-group associations and frequency of various types of incidents.

## Results

In total, 93 male patients were discharged from the hospital during the data collection period; 9 patients were excluded from the study: 7 were excluded as their admission was less than 3 months and unlikely to be representative of the treatment phase being evaluated, and discharge would also have occurred prior to the standard Care Programme Approach meeting held 3 months after admission, where a formal diagnosis would have been made. One patient was excluded as they were informal during the course of their admission and one was excluded due to death from natural causes. Therefore, 84 patients were included in the study, with 32 in the civil group and 52 in the forensic group. In the civil group, 16 patients were admitted from general adult services, 1 from a police station, 7 from low secure services and 8 from medium secure services. The legal status of patients in the civil group remained unchanged during the course of their admission, apart from one patient who became informal in the days prior to discharge. None of the patients in the civil group switched to being forensic patients following convictions in court. Regarding the forensic group, 4 patients were admitted from general adult services, 3 from low secure services, 16 from medium secure services, 28 from prison and 1 from a high secure hospital. In this group, 14 patients changed their legal status prior to discharge; 11 changed from being sentenced prisoners under Section 47/49 to being detained under a notional Section 37, as they had gone past what would have been their automatic release date from prison. Two patients switched from being remanded prisoners under Section 48/49 to being sentenced under a Section 37 hospital order at court and one patient switched from Section 48/49 to a Section 37/41 hospital order with restrictions after sentencing at court.

Table 1 outlines the baseline characteristics of each group, including diagnosis, age, ethnicity and Mental Health Act status on admission. All patients were male, with a mean age of 37 years (range 20 to 63 years). Table 2 shows the mean length of stay, HoNOS-secure scores on admission and discharge, and frequency of risk-related incidents.

**Table 1:** Patient characteristics

	Civil group^a^ *n* (%)	Forensic group^b^ *n* (%)
Primary diagnosis		
Psychosis (schizophrenia, schizoaffective disorder, delusional disorder)	30 (94)	46 (88)
Personality disorder	2 (6)	3 (6)
Affective disorder (depression, bipolar affective disorder)	0 (0)	3 (6)

Secondary diagnosis		
Personality disorder	5 (16)	11 (21)
Substance misuse	18 (56)	33 (63)
Alcohol misuse	1 (3)	7 (13)

Ethnicity		
Black	10 (31)	13 (25)
White	18 (56)	27 (52)
Other	4 (13)	12 (23)

Legal status		
Section 2	1 (3)	
Section 3	31 (97)	
Section 37		8 (15)
Section 47 (notional 37)		5 (10)
Section 37/41		12 (23)
Section 48/49		7 (13)
Section 47/49		20 (39)

a. *n*=32.

b. *n*=52.

**Table 2:** Length of stay in secure care and frequency of risk-related incidents

	Civil group^a^	Forensic group^b^	Independent *t*-test^c^
Length of stay, days: mean (range)	587 (95–1396)	523 (105–1407)	*t* = 0.75, *P* = 0.96

Mean HoNOS-secure score:			
admission	25.31	24.62	*t* = −0.39, *P* = 0.07
discharge	20.16	18.77	*t* = −0.81, *P* = 0.94

Risk incidents per 30 days, mean			
Violence (includes assaults against staff or peers)	0.92	0.34	*t* = 2.01, *P* = 0.02
Self-harm (threats or acts)	0.06	0.21	*t* = −2.09, *P* = 0.02
Unauthorised leave (attempts or episodes of absconding or escape)	0.22	0.05	*t* = 1.17, *P* = 0.44
Substance misuse (intentions or incidents of illicit drug misuse)	0.06	0.12	*t* = −1.14, *P* = 0.15
Self-neglect (poor self-care/diet)	0.41	0.25	*t* = 1.39, *P* = 0.17
Fire-setting (threats or acts)	0.08	0.02	*t* = 1.76, *P* = 0.002
Sex offending (sexual comments or contact offences)	0.06	0.03	*t* = 1.09, *P* = 0.04
Vulnerability (being intimidated, bullied or assaulted)	2.10	0.29	*t* = 4.88, *P* = 0.00
Verbal aggression (abusive comments)	2.10	1.62	*t* = 0.87, *P* = 0.36
Other unspecified risk incidents	1.85	1.99	*t* = −0.33, *P* = 0.36

a. *n*=32.

b. *n*=52.

c. d.f=82.

## Discussion

The study found no significant difference in length of stay or severity of illness based on HoNOS-secure scores at the start or end of admission between the civil and forensic groups. However, it should be noted that HoNOS-secure is not a specific measure of mental state, since it also evaluates behavioural functioning and a range of security measures. This study identified that civil patients in secure settings have more frequent incidents of aggression, sex offending, fire-setting and vulnerability, whereas forensic patients have more frequent episodes of self-harm. This finding challenges the preconception that forensic patients are more ‘dangerous’ and difficult to manage.^2^ One explanation for this may be that the civil patients in this study represent a cohort of general adult patients that have been placed in forensic services due to their frequency of aggressive and difficult to manage behaviours, whereas the more stable forensic patients have been admitted due to severe but more isolated offences.

The higher frequency of incidents in the civil group may make engaging with specialist treatment programmes practically more difficult for this group, which may in turn become a factor that limits their motivation to engage. Secure hospitals should be aware that civil patients, due to higher frequency of risk incidents, may have differing needs to forensic patients. Therefore, we suggest that civil patients who present with a high frequency of incidents will benefit from a greater emphasis on treatment of their mental illness combined with behavioural interventions, with less of a requirement to engage in specialist treatment programmes or to complete formal psychological therapy programmes that are often required in forensic settings. Further research is needed to explore whether the higher frequency of incidents among civil patients affects the therapeutic milieu on the ward and has an adverse impact on outcomes for forensic patients engaging in specialist treatment interventions. The higher frequency of vulnerability incidents among civil patients highlights the difficulty they experience in forensic settings and suggests a greater need for vigilance and robust safeguarding for this patient group, who may be at risk of reprisal assaults by their forensic peers. The findings of our study must also be considered in light of the Schizophrenia Commission report,^7^ which comments that patients stay too long in secure services, and highlights funding cuts and acute bed closures in general adult services as part of the problem.

We conclude that our study supports the need to focus more on preventive interventions, such as avoiding delays in assessment, ensuring early treatment and supporting alternatives to admission such as crisis and home-based treatment teams, to help avoid admissions. Regular liaison between forensic and general adult services is essential to help ensure patients can return to appropriate settings at the earliest opportunity in their recovery. This may only be possible with careful consideration when commissioning services at all levels of care.

The finding that forensic patients have a greater frequency of self-harm incidents should be treated with caution as the numbers in this study are small and self-harm is a rare outcome. One possibility is that forensic patients may find the criminal justice system and their conviction distressing, leading to a greater risk of self-harm and potentially suicide. We suggest that clinical teams should be aware of this risk in these patients and ensure careful monitoring, risk management and support for patients during criminal proceedings.

### Limitations

This study has a number of limitations. Most significantly, it is a comparison of forensic and civil patients conducted in a secure mental health hospital and the findings cannot be used to compare differences between forensic and general adult patients in non-secure settings. In addition, the civil patients in the study are likely to represent patients with greater treatment resistance whose aggressive behaviours have led to them being transferred to secure settings. It remains possible that the section status assigned to the patient on admission may be misleading, as quite often patients who commit offences when unwell are not prosecuted.^8^ The study is reliant on accurate recording of risk incidents in patients' records. Although some degree of inaccuracy in recording of incidents may have occurred, it is anticipated that this would have occurred evenly between both groups and thus not affected the validity of the results. This study, in line with previous work, evaluates data for a cohort of discharged patients in order to evaluate comparable groups. It is possible that the study may underestimate the severity of risk incidents, since the most challenging patients would not have been included in the analysis as they have not yet been discharged from hospital. It is anticipated that the impact of this factor would be evenly distributed between each group.

### Practice recommendations

Secure hospitals should ensure all treatment plans are based around the individual. There should be an emphasis on managing the mental illness of civil patients and tailoring treatments based on this goal, which will help reduce risks and hopefully shorten length of admission. Forensic patients are more likely to have additional offence-related treatment needs which would require specific interventions. Regular liaison between forensic and general adult services is essential to help ensure patients can return to appropriate settings at the earliest opportunity in their recovery. This can only be possible with careful consideration when commissioning services at all levels of care.
